# Inducible Genome Editing with Conditional CRISPR/Cas9 Mice

**DOI:** 10.1534/g3.117.300327

**Published:** 2018-03-08

**Authors:** Alexandra Katigbak, Francis Robert, Marilène Paquet, Jerry Pelletier

**Affiliations:** * Department of Biochemistry, Faculté de Médecine Vétérinaire, Université de Montréal, Saint-Hyacinthe, Québec; ‡ The Rosalind and Morris Goodman Cancer Research Center, McGill University, Montreal, Québec, Canada, H3G 1Y6; § Department of Oncology, McGill University, Montreal, Québec, Canada, H3G 1Y6; † Département de Pathologie et Microbiologie, Faculté de Médecine Vétérinaire, Université de Montréal, Saint-Hyacinthe, Québec

**Keywords:** CRISPR/Cas9, genome editing, mouse model, Cas9 knock-in, conditional Cas9 mouse

## Abstract

Genetically engineered mouse models (GEMMs) are powerful tools by which to probe gene function *in vivo*, obtain insight into disease etiology, and identify modifiers of drug response. Increased sophistication of GEMMs has led to the design of tissue-specific and inducible models in which genes of interest are expressed or ablated in defined tissues or cellular subtypes. Here we describe the generation of a transgenic mouse harboring a doxycycline-regulated Cas9 allele for inducible genome engineering. This model provides a flexible platform for genome engineering since editing is achieved by exogenous delivery of sgRNAs and should allow for the modeling of a range of biological and pathological processes.

Rapid and facile genome editing has been enabled through the use of type II bacterial CRISPR (clustered, regularly interspaced, short palindromic repeats)/Cas9 (CRISPR-associated protein) systems. By taking advantage of RNA-directed targeting, the Cas9 endonuclease is used to induce DNA breaks at a given locus. These are subsequently repaired by either the mutagenic NHEJ (non-homologous end joining) pathway, by HDR (homology-directed recombination) if a repair template complementary to the targeted region is available, or HITI (homology-independent targeted integration) in which a transgene becomes inserted at a double-stranded cleaved site (Suzuki *et al.* 2016). This game changing technology has been used in a myriad of applications; ranging from *ex vivo* and *in vivo* genome editing to the rapid development of novel animal models for disease.

To extend the utility of CRISPR/Cas9 for *in vivo* functional studies, transgenic mice expressing Cas9 in their germline have been developed. A Cre-dependent Cas9 knock-in mouse in which Cas9 expression is activated in a tissue-specific manner has been used to model lung adenocarcinoma by simultaneously inactivating *p53* and *Lkb1* by NHEJ mutagenic repair, while generating *Kras^G12D^* alleles by HDR (Platt *et al.* 2014). Another Cas9 transgenic mouse strain has been built in which a Cre/loxP-dependent conditional Cas9 allele was engineered into the *Rosa26* locus (Chu *et al.* 2016). Using a different strategy, Dow *et al.* (Dow *et al.* 2015) produced GEMMs (genetically engineered mouse models) co-expressing DOX (doxycycline)-inducible Cas9 and a single guide (sg) RNAs in their germline. This latter platform illustrated the feasibility of inducible *in vivo* genome editing at multiple loci (*p53* and *Apc*) to model cancer progression (Dow *et al.* 2015). These powerful models are enabling the application of Cas9 editing technology to a number of tissue- and embryo-based settings.

Here, we report on the generation of a DOX-inducible Cas9 mouse in which we placed a TRE (tetracycline responsive element)-inducible Cas9 allele into the *Col1A1* locus. This mouse overcomes the *in vivo* delivery challenges of Cas9, avoids potential genotoxicity associated with *Cre* recombinase (Loonstra *et al.* 2001), and maintains flexibility with respect to choice of sgRNA delivery.

## Materials And Methods

### Generating Col1A1 Knock-in Cas9 Mice

A pUC57 derivative (pUC57a) with appropriate linker sequences tailored for multi-component assembly of the donor template was purchased from GenScript and contained the following adaptor sequence: ^5′^CCATGGTGATGCATATGGCCGTGAAGAGACCCGCCGCCACCAAGAAGGCCGGCCTTAATTAAACGCGTTGAGAACTTCAGGGTGAGTTTGGGGACCCTTGATTGTTCTTTCTTTTTCGCTATTGTAAAATTCATGTTATATGGAGGGGGCAAAGTTTTCAGGGTGTTGTTTAGAATGGGAAGATGTCCCTTGTATCACCATGG^3′^. Using unique *Fse*I/*Pac*I (NEB; New England Biolabs) restriction sites, the Flag-Cas9-IRES-GFP fragment from pQCiG2 (Malina *et al.* 2014) was cloned into pUC57a. The GFP ORF was transferred from pUC57a-Cas9-IRES-GFP into pCol-TGM-p53.1224 (Premsrirut *et al.* 2011) using *Nco*I (NEB). The resulting plasmid was partially cleaved with *Nco*I and the Cas9-IRES fragment from the parental pUC57a-Cas9-IRES-GFP vector transferred to generate pCol-Tre-Cas9-iG. Unique *Asc*I/*Xmn*I restriction sites were then used to transfer the CAGs-rtTA3-SAdpA cassette (Dow *et al.* 2014) into pCol-Tre-Cas9-iG, downstream of GFP to generate the knock-in donor template, pCol-TCiG-rtTA3 (Figure S1). Plasmids are available from the authors upon request.

C10 ES cells were cultured in complete knock-out DMEM (15% ESC Qualified Serum, 1% Penicillin-Streptomycin, 1% Non-Essential Amino Acids, 1% L-Glutamine, 0.1% BME, 0.01% LIF) on gelatinized plates with PMEF-N Feeders (Millipore). Fifty micrograms of pCol-TCiG-rtTA3 was electroporated with 25 µg of Flpe recombinase expression plasmid (pCAGs-Flpe) as previously described (Lin *et al.* 2012). After 2 days of recovery, recombinant clones were selected using 140 µg/mL hygromycin, assessed for DOX inducibility, and used to generate chimeras. The TRE-Cas9 allele has been crossed onto the C57BL/6 background for >6 generations. All animal studies were approved by the McGill University Faculty of Medicine Animal Care Committee.

### PCR and Genotyping

Cas9 allele status was assessed with primers Col1A1-F: ^5′^AATCATCCCAGGTGCACAGC^3′^, SAdpA-R (from Mirimus, NY): ^5′^CTTTGAGGGCTCATGAACCTCCCAGG^3′^, and Col1A1-R: ^5′^ACCGCGAAGAGTTTGTCCTCAAC^3′^. Primers Col1A1-F and Col1A1-R provided a characteristic 379 bp band indicative of the presence of Cas9, whereas Col1A1-F and SAdpA-R generated a 239 bp band indicative of a wt *Col1A1* locus. The *Rosa26* locus status was assessed using the following primers: Rosa-A: ^5′^AAAGTCGCTCTGAGTTGTTAT^3′^, Rosa-B: ^5′^GCGAAGAGTTTGTCCTCAACC^3′^, Rosa-C: ^5′^GGAGCGGGAGAAATGGATATG^3′^. Rosa-A and Rosa-B produce a ∼500 bp band, indicative of a wild-type *Rosa26* allele, while Rosa-A and Rosa-C produce a ∼300 bp band, indicating the presence of the rtTA allele. Eµ-Myc allele status was assessed using primers 5′Eµ-Myc: ^5′^GGACAGTGCTTAGATCCAAGGTGA^3′^, and 3′Eµ-Myc: ^5′^CCTCTGTCTCTCGCTGGAATTACT^3′^ which produces a 600 bp band when the Eµ-Myc allele is present. Recovery of the sgp53-3 sequences from tumor samples was achieved by PCR amplification using primers ^5′^GAAGATCTTCTAGAGATCCG^3′^ and ^5′^AAAAAGCACCGACTCGGTGC^3′^ (Cencic *et al.* 2014).

### Construction of the pUSPPC sgRNA-expression Vector

To generate pUSPPC, we first replaced GFP in pQCiG2 with mCherry by digesting with *Eco*RV/*Cla*I to remove the IRES-GFP sequence. This was replaced with the EMCV IRES which was PCR amplified from pQCiG2 using primers “IRES-F” ^5′^AGTACGTAGATATCCCCATTAATCGATTTGAATTCCG^3′^ and “IRES-R” ^5′^AGTACGTAATCGATACTAGTGTGGCCATATTATCATCG^3′^ and digested with *Cla*I/*Eco*RV and ligated into the gutted pQCiG2 vector to produce ‘pQCi’. The mCherry coding region sequence was amplified using PCR with primers “mCherry-F” ^5′^ATATCGCCTAGGCTTTTGCAAAAAGC^3′^ and “mCherry-R” ^5′^ATATCGCCTAGGTTACTTGTACAGCTCGTCCATG^3′^ with Vent polymerase. This amplicon was then digested with *Avr*II and cloned into pQCi, which had been linearized with *Spe*I, to form ‘pQCiC’. The Cas9 expression cassette was then removed from this vector using unique *Xho*I/*Eco*RV sites. The PGK-Puromycin cassette from pPrime-shRNA (Robert *et al.* 2014) was excised by first digesting with *Pac*I, repairing with T4 DNA polymerase, and cleaving with *Xho*I. The resulting product was ligated into pQCiC vector to generate pUSPPC (Figure S2).

### T7 Endonuclease I (T7EI) Cleavage Assay

Genomic DNA from pUSPPC-transduced HSPCs was prepared using a Zymo Research Quick-gDNA MiniPrep kit (D3006). PCR amplification of the sgp53-3 targeted region of *Trp53* was performed using Primer p53-3F:^5′^CCTGATCGTTACTCGGCTTGT^3′^ and Primer p53-3R: ^5′^CAAGAATAAGTCAGAAGCCGGG^3′^ using Phusion High-Fidelity polymerase. The T7EI assay was then performed as previously described (Malina *et al.* 2013) and the entire reaction resolved on a 15% 1x TBE polyacrylamide gel (29:1 acrylamide:bisacrylamide) before staining with SybrGold (ThermoFisher).

### HSPC Adoptive Transfers

Low passage Phoenix-Eco packaging cells were cultured in complete DMEM (10% FBS, 1% Penicillin-Streptomycin, 1% L-Glutamine) and grown at 37°/5% CO_2_. Twenty four hours prior to transfection, 3.5 × 10^6^ Phoenix-Eco cells were seeded in 10 cm^2^ tissue culture plates. Plasmids (10 µg) were co-transfected with 1µg pCL-Eco replication-incompetent helper vector using calcium phosphate (Naviaux *et al.* 1996). Twenty-four hours after transfection and 12h before the first virus harvest infection, plates were washed with PBS and refreshed with 5 mL complete BCM (45% DMEM, 45% IMDM 10% FBS, 1% Penicillin-Streptomycin, 1% L-Glutamine). Twelve hours after refreshing media, virus was collected every 12h for a period of 48h.

R26-rtTA;TRE-CiG/rtTA;Eµ-Myc HSPCs were isolated from fetal livers at E13.5 days as previously described (McCurrach and Lowe 2001). Cells were placed in culture 12 hr before infection in BCM supplemented with 1 ng/mL IL-3, 10 ng/mL IL-6, 100 ng/mL SCF and incubated at 37°/5% CO_2_. Cultured HSPCs were infected four times at 12h intervals with viral supernatant from transfected Phoenix-Eco cells, supplemented with 1 ng/mL IL-3, 10 ng/mL IL-6, 100 ng/mL SCF and 4 µg/mL polybrene, and spinoculated at 950 xg for 1 hr at 32°. To induce Cas9 expression, cells were treated *ex vivo* with 1µg/mL doxycycline. Transduction and GFP-induction efficiency was assessed by flow-cytometry prior to transplantation (Guava EasyCyte 8HT, Millipore).

For transplantations, 6-8 week old female C57BL/6 mice were placed on 0.125 mg/mL ciprofloxacin/2% sucrose two days before transplantation. Four hours before transplantation, mice were irradiated with 4 Gy γ radiation. Approximately 6 × 10^5^ HSPCs were transplanted into irradiated mice by intravenous tail-vein injection 24 hr after the last transduction. Mice were palpated bi-weekly to assess tumor status until the experimental end point at day 120.

### Western Blots

Extracts were prepared from frozen cell pellets. Pellets were resuspended in RIPA buffer (20 mM Tris-HCl [pH 7.5], 150 mM NaCl, 0.1% SDS, 1% NP40, 0.5% sodium deoxycholate, 1 mM β-glycerophosphate, 1 mM PMSF, 1 μg/ml leupeptin, 10 μg/ml aprotinin, and 2.5 μM pepstatin) and incubated on ice for 10 min. Lysates were denatured in Laemmli sample buffer by heating to 90° for 10 min. Proteins were resolved on 10% NuPAGE gels and transferred to PVDF membranes by electroblotting at 200 mA/gel for 2 hr. Antibodies used were: α-FLAG (1:5000, Sigma), α-Cas9 (1:1000, Abcam ab191468), α-GAPDH (1:1000, Abcam ab8245), α-eEF2 (1:1000, Cell Signaling 2332).

### Southern Blot Analysis of the Col1A1 Locus

Genomic DNA was isolated from ES cell clones, digested with *Eco*RI, and fractionated on a 0.8% TBE agarose gel (Sambrook and Russell 2001). After transfer to Hybond N+ membranes, the DNA was interrogated using a probe targeting the hygromycin gene outside of the region targeted by the donor template. The probe was generated by PCR amplification using primers A (^5′^ATGAAAAAGCCTGAACTCACCG^3′^) and B (^5′^CCAATGTCAAGCACTTCCG^3′^) and labeled using ThermoFisher DecaLabel DNA Labeling Kit (K0662) with α-^32^P-dCTP (New England Nuclear, MA). Following hybridization, membranes were washed in 2x SSC/0.1% SDS once at 25°, twice at 55°, and then once with 1x SSC/0.1% SDS at 55°.

### Immunophenotyping and Immunohistochemical Analysis

Spleens were harvested from mice, macerated, and passed through a 40 µm cell strainer to create single-cell suspensions. Red blood cells were eliminated by lysis in ACK lysis buffer (150 mM NH_4_Cl, 10 mM KHCO_3_, 0.1 mM EDTA) [pH 7.2] for 5 min on ice, before neutralizing with PBS. 300,000 cells were stained either with 0.06 µg PE-conjugated **α**-CD4 (BD Pharmigen 553652) or **α**-B220 (BD Pharmigen 553090) for 30 min before washing with PBS. PE^+^ and GFP^+^ populations were analyzed by flow cytometry (Guava EasyCyte 8HT, Millipore).

Tissues were harvested from mice and fixed in 10% buffered formalin for 48 h before embedding in paraffin. Sections (4 µm) were de-paraffinized using xylene and rehydrated through a series of decreasing concentration of ethanol washes, followed by a final water wash. Antigen retrieval was performed in a pressure cooker for 15 min in 10 mM sodium citrate (pH 6.0)/ 0.05% Tween-20. After washing, samples were blocked using TBS [pH 7.5]/ 10% FBS/ 1% BSA for 2h at RT and incubated with α-GFP antibodies (1:500, Cell Signaling 2555) overnight at 4°. Slides were blocked with H_2_O_2_ for 10 min, incubated for 30 min with biotinylated goat anti-rabbit IgG and then streptavidin peroxidase (Anti-rabbit HRP/DAB detection kit, Abcam). Staining was performed using DAB chromogen and substrate from Abcam and counterstained using IHC-optimized hematoxylin (Vector Labs). Sections were dehydrated, mounted using permount, and slides scanned using an Aperio XT slide scanner with the resulting images analyzed using Aperio ScanScope.

### Data availability

The authors state that all data necessary for confirming the conclusions presented in the article are represented fully within the article.

## Results

### Generation of a Doxycyline (DOX)-Inducible Cas9 Mouse

**Figure 1:**
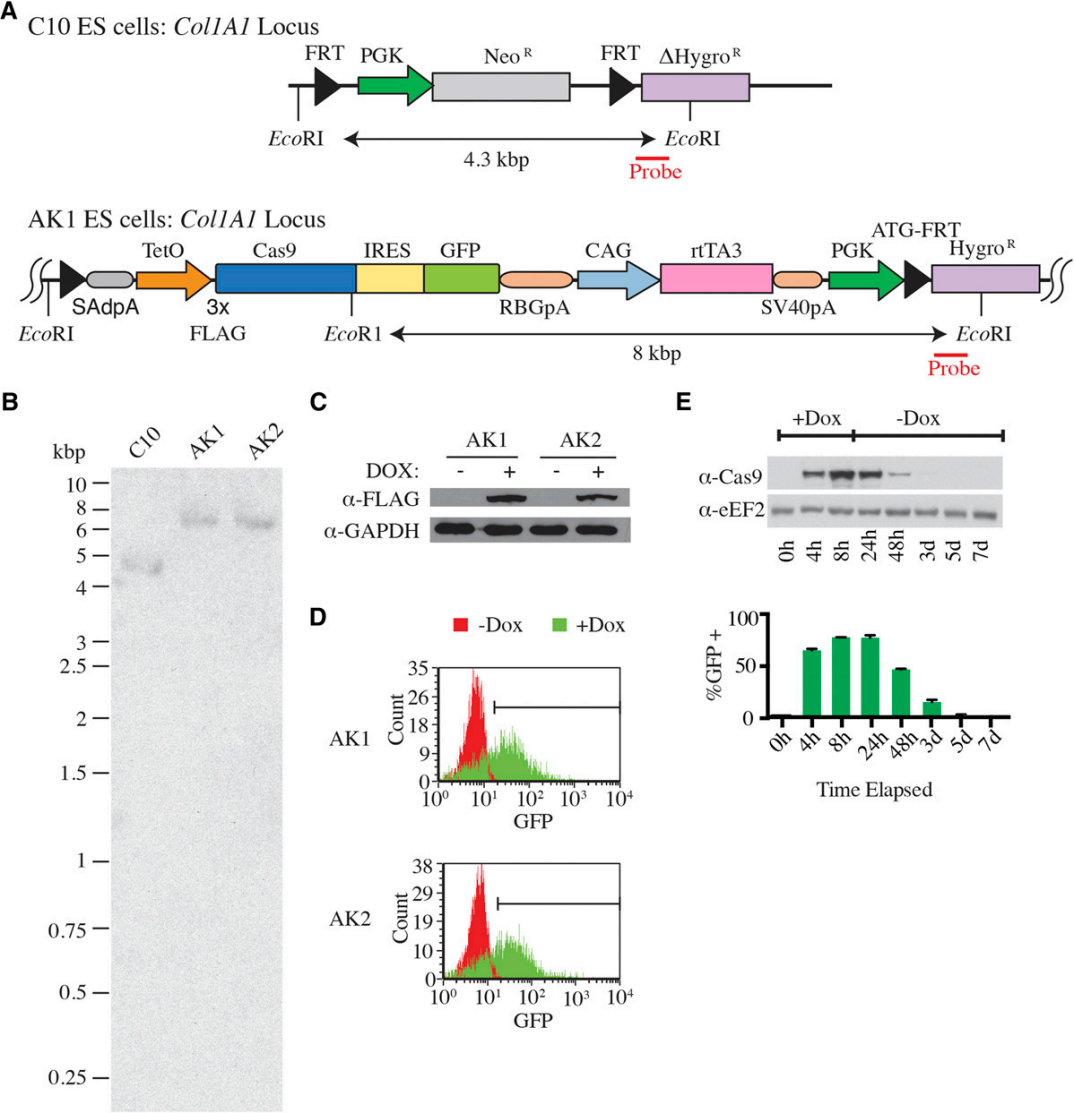
Inducible and reversible Cas9 expression in murine ESCs. A. Configuration of the *Col1A1* locus in C10 ESCs (top) as well as following Flpe-mediated recombination (bottom). The diagnostic *Eco*R1 sites and the region outside of the targeting vector used for probe generation (red bar) to confirm integration by Southern blotting are indicated. FRT, Flippase recombinase target; PGK, phosphoglycerate kinase promoter; Neo^R^, aminoglycoside phosphotransferase, neomycin resistance protein; ΔHygro^R^, hygromycin B phosphotransferase, ATG codon lacking; SAdpA, splice acceptor & donor polyadenylation signal cassette; TetO, tetracycline operators; FLAG, DYKDDDDK epitope tag; IRES, internal ribosome entry site; RBGpA, rabbit β-globin polyadenylation signal; CAG, cytomegalovirus enhancer fusion to chicken β-actin promoter; rtTA3, reverse tetracycline-controlled transactivator, version 3; SV40pA, simian virus 40 polyadenylation signal; ATG-FRT, ATG containing flippase recombinase target. B. Southern blot analysis of the parental C10 ES cell line and two hygromycin-resistant clones, AK1 and AK2, using a probe downstream and external to the FRT site (see panel A, red bar). C. Western blot illustrating Cas9 induction in ESC clones 48 h following 1 ug/ml DOX treatment. Blots were probed with the indicated antibodies. D. GFP induction in ESC clones 48 h following 1 μg/ml DOX treatment as assessed by flow cytometry. E. Western blot illustrating reversible Cas9 expression in AK1 cells. AK1 cells were treated with 1 μg/ml DOX for 24 h, after which time fresh media lacking DOX was added. At the indicated time points, aliquots of cells were taken for Western blotting and analysis by flow cytometry.

Recombinase-mediated cassette exchange (RMCE) is a rapid method by which to generate transgenic mice with DOX-inducible cDNAs or shRNAs. This approach is facilitate by the existence of pre-engineered C10 ES cells containing a FRT-hygro-pA “homing” cassette downstream of the *Col1A1* locus (Beard *et al.* 2006; Premsrirut *et al.* 2011) (Figure 1A, top panel). We took advantage of the ease of manipulation of these cells and used FLPe recombinase to mediate recombination between the FRT sites at the *Col1A1* locus and a site present in our pCol-TCiG-rtTA3 targeting vector (Beard *et al.* 2006; Premsrirut *et al.* 2011). In this vector, we placed Cas9 and GFP under regulation of the tetracycline response elements (TRE) and positioned a second transcriptional unit downstream with the CAGs promoter (Okabe *et al.* 1997) driving reverse tet-transactivator (rtTA3) expression (Figure 1A, bottom panel). Following ES cell electroporation and RMCE, two hygromycin resistant cells, AK1 and AK2, were clonally expanded and characterized. We first ensured correct integration at the *Col1A1* locus by Southern blotting, which revealed a diagnostic 8 kbp *Eco*RI fragment (Figures 1A, B). Both AK1 and AK2 ES cells showed DOX-dependent induction of Cas9 and GFP expression (Figures 1C, D). Expression of both Cas9 and GFP was reversible in AK1, with little Cas9 left 3 days after removal of DOX from the culture media (Figure 1E).

### Characterization of Inducible Cas9 Expression in Mice

**Figure 2:**
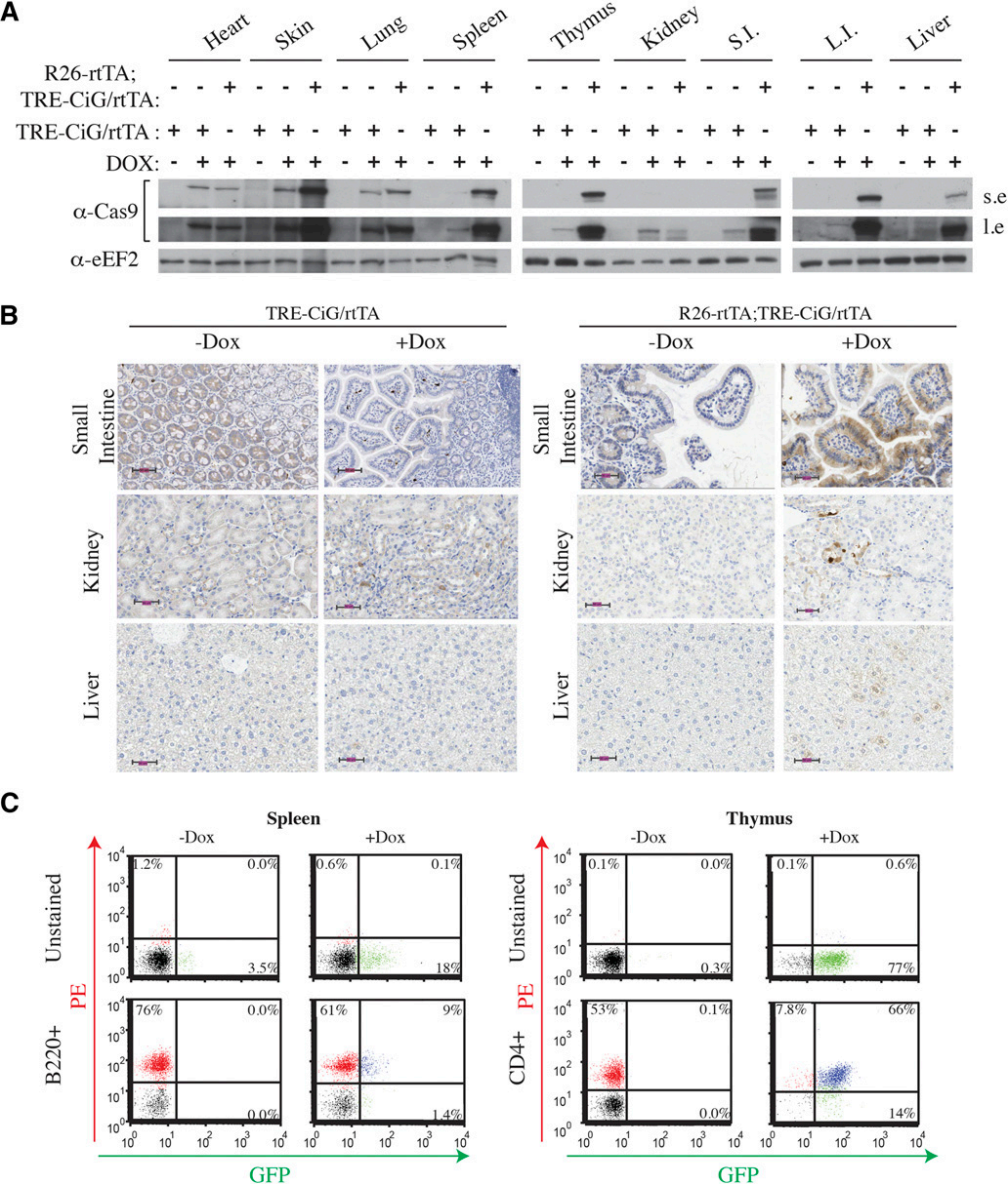
Assessing induction of Cas9 expression *in vivo*. A. Western blot of Cas9 from the indicated tissues harvested from TRE-CiG/rtTA or R26-rtTA;TRE-CiG/rtTA mice treated with vehicle (-) or DOX (+) for 1 week. S.I., small intesting; L.I, large intestine; s.e., short exposure; l.e., long exposure. B. Immunohistochemical staining for GFP expression in the small intestine, kidney, and liver from the indicated mice (+/− DOX). Magnification bars denote 50 μm. C. Quantitation of B220+ (pan B cell marker) and CD4+ (T) cells isolated from the spleen and thymus, respectively, of R26-rtTA;TRE-CiG/rtTA mice that had been treated with DOX for 1 week.

Transgenic mice were produced from AK1 ESCs, referred to henceforth as TRE-CiG/rtTA, and their preliminary characterization revealed weak global GFP induction in a large number of tissues following DOX treatment (data not shown). We reasoned that one possibility for this could be limiting rtTA3 activity and/or levels. Indeed, DOX induction of the TRE promoter *in vivo* can be restricted by limiting rtTA levels – as documented in GEMMs harboring conditional shRNAs (McJunkin *et al.* 2011). Specifically, McJunkin *et al.* (McJunkin *et al.* 2011) demonstrated that shRNA-mediated suppression of Replication Protein A (subunit 3) (Rpa3) *in vivo* is more potent when two rtTA expressing alleles are present in the germline of shRpa3-bearing mice, compared to mice expressing only one rtTA allele (McJunkin *et al.* 2011). To assess if rtTA was limiting in our system, we crossed Rosa26(R26)-rtTA mice to our TRE-CiG/rtTA GEMM and found that the resulting R26-rtTA;TRE-CiG/rtTA offsprings displayed higher induced levels of Cas9 and GFP in a number of analyzed tissues compared to TRE-CiG/rtTA mice following DOX exposure (*e.g.*, skin, spleen, thymus, small and large intestine, liver) (Figures 2A, B). Induction of GFP was also observed in B cells isolated from spleen and thymus of R26-rtTA;TRE-CiG/rtTA mice (Figure 2C, 9%). The low level of induction in B cells may indicate that SpCas9 and GFP expression are uncoupled in this cell type.

**Figure 3:**
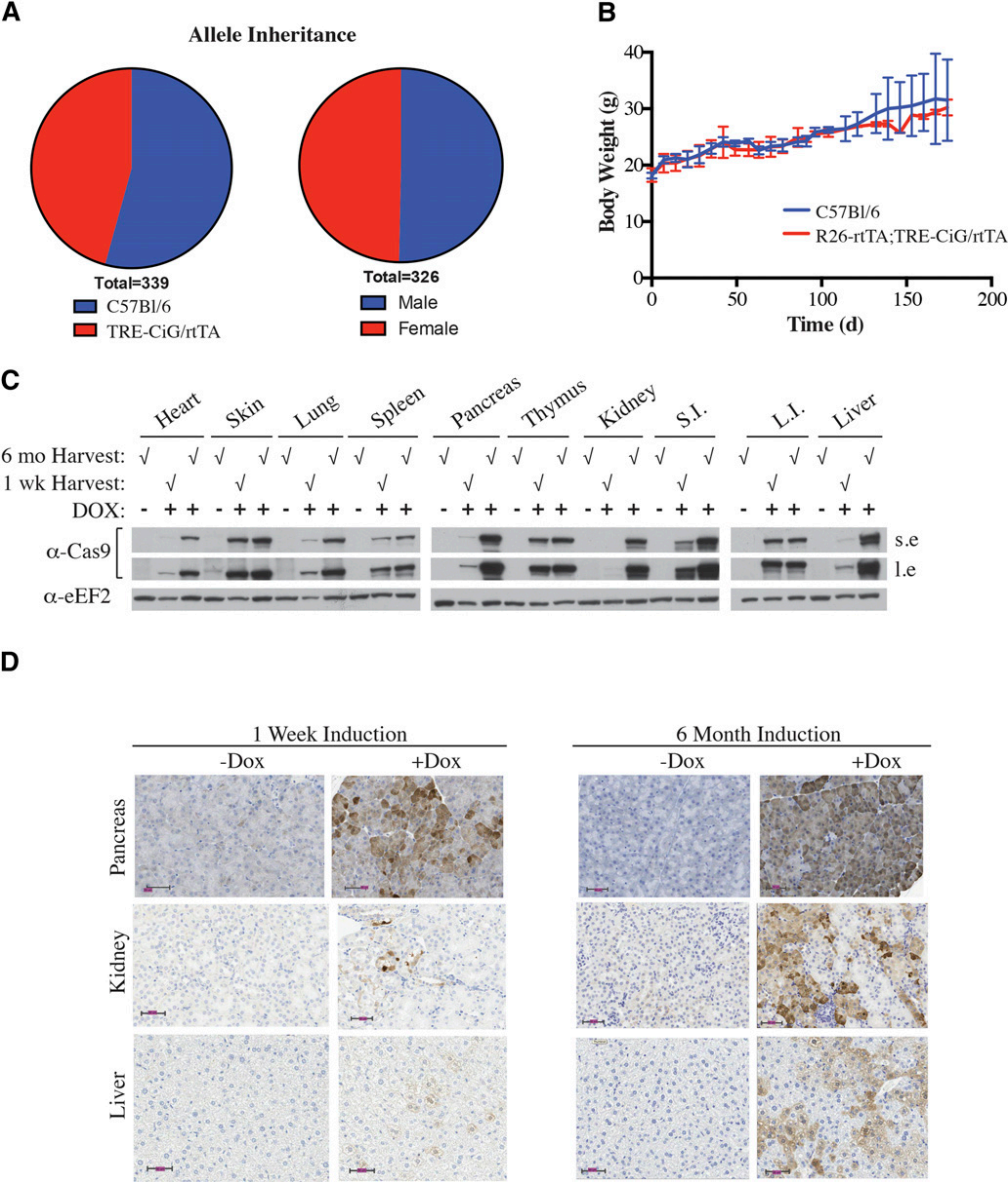
Expression of Cas9 is well tolerated in mice. A. Mendelian inheritance frequency of the TRE-CiG/rtTA allele, as well as sex distribution of the inheritance frequency. B. Body weight of mice that had been treated with DOX for 6 months. N= 2, +/− error of the mean. C. Western blot of Cas9 from the indicated tissues harvested from R26-rtTA;TRE-CiG/rtTA mice treated with vehicle (-) or DOX (+) for 1 week (wk) or 6 months (mo). D. Immunohistochemical analysis of GFP expression in the pancreas, kidney, and liver from R26-rtTA;TRE-CiG/rtTA mice exposed to vehicle or DOX for 1 week (left) or 6 months (right). Magnification bars denote 50 μm.

We did not notice any evidence of toxicity associated with expression of the Cas9 transgene. Mice harboring the TRE-CiG/rtTA allele were fertile, had normal litters, and appeared morphologically normal. The TRE-CiG/rtTA allele was inherited at the expected Mendelian frequency with no significant associated sex effects in the inheritance pattern (Figure 3A). Long-term (6 months) treatment of R26-rtTA;TRE-CiG/rtTA mice with DOX did not affect weight gain (Figure 3B) nor overall general behavior. Cas9 was still expressed in tissues of mice continuously receiving DOX for 6 months and in most cases, levels appeared even higher than in tissues from R26-rtTA;TRE-CiG/rtTA mice that had been on DOX for 1 week (Figure 3C). Tissue analysis showed no discernible histological changes and we found no evidence of increased apoptosis (Figure 3D and data not shown). Additionally, blood chemistry from R26-rtTA;TRE-CiG/rtTA mice after 6 months of DOX treatment showed almost all values within normal range, with the values of blood cholesterol and potassium being the only parameters slightly outside the norm (Table 1). This is consistent with what has been reported for mice constitutively expressing Cas9 (Platt *et al.* 2014) in that long-term sustained Cas9 expression is not associated with any overt detrimental phenotype or negative impact on the animal’s well being.

**Table 1:** Blood Chemistry Analysis of R26-rtTA;TRE-CiG/rtTA Mice 6 Months Post-Induction

**Criterion**	**Units**	**Normal Range**	**R26-rtTA;TRE-CiG/rtTA (n = 2)**
Total Protein	g/L	31-66	46
Albumin	g/L	25-48	22
Albumin/Globulin Ratio			0.9
Glucose	mmol/L	5.0-10.7	9.9
BUN Urea	mmol/L	6.4-10.4	7.05
Creatinine	µmol/L	18-71	18
Total Bilirubin	µmol/L	2-15	3
ALT	U/L	28-132	31.5
AST	U/L	59-247	78
Alkaline Phosphatase	U/L	62-209	115
CK	U/L	68-1070	136
Cholesterol	mmol/L	0.93-2.48	2.56
Sodium	mmol/L	124-174	50.5
Potassium	mmol/L	4.6-8.0	4.28
Chloride	mmol/L	92-120	115
Calcium	mmol/L	1.47-2.35	2.105
Phosphorus	mmol/L	1.97-3.26	2.205
Magnesium	mmol/L	0.33-1.60	1.06

### Ex Vivo Genome Editing in Primary Hematopoietic Stem and Progenitor Cells (HSPCs)

The Eµ-Myc mouse model is a robust and malleable model of non-Hodgkin’s lymphomas (Harris *et al.* 1988). Very powerful genetic screens for novel oncogenic drivers have utilized the Eμ-Myc model, capitalizing on the ability to manipulate HSPCs derived from embryos *ex vivo* coupled with *in vivo* selection. For example, the approach has identified novel oncogenic drivers by infecting Eμ-Myc HSPCs with libraries of shRNAs followed by transplantation into normal recipients and monitoring for tumor onset (Scuoppo *et al.* 2012). Recently, we have taken advantage of this model and employed an *in vivo* CRISPR/Cas9 screen to identify and distinguish rare oncogenic modifier events from passenger or bystander mutations identified in human Burkitt’s lymphoma whole genome, exome, and transcriptome data (Katigbak *et al.* 2016).

**Figure 4:**
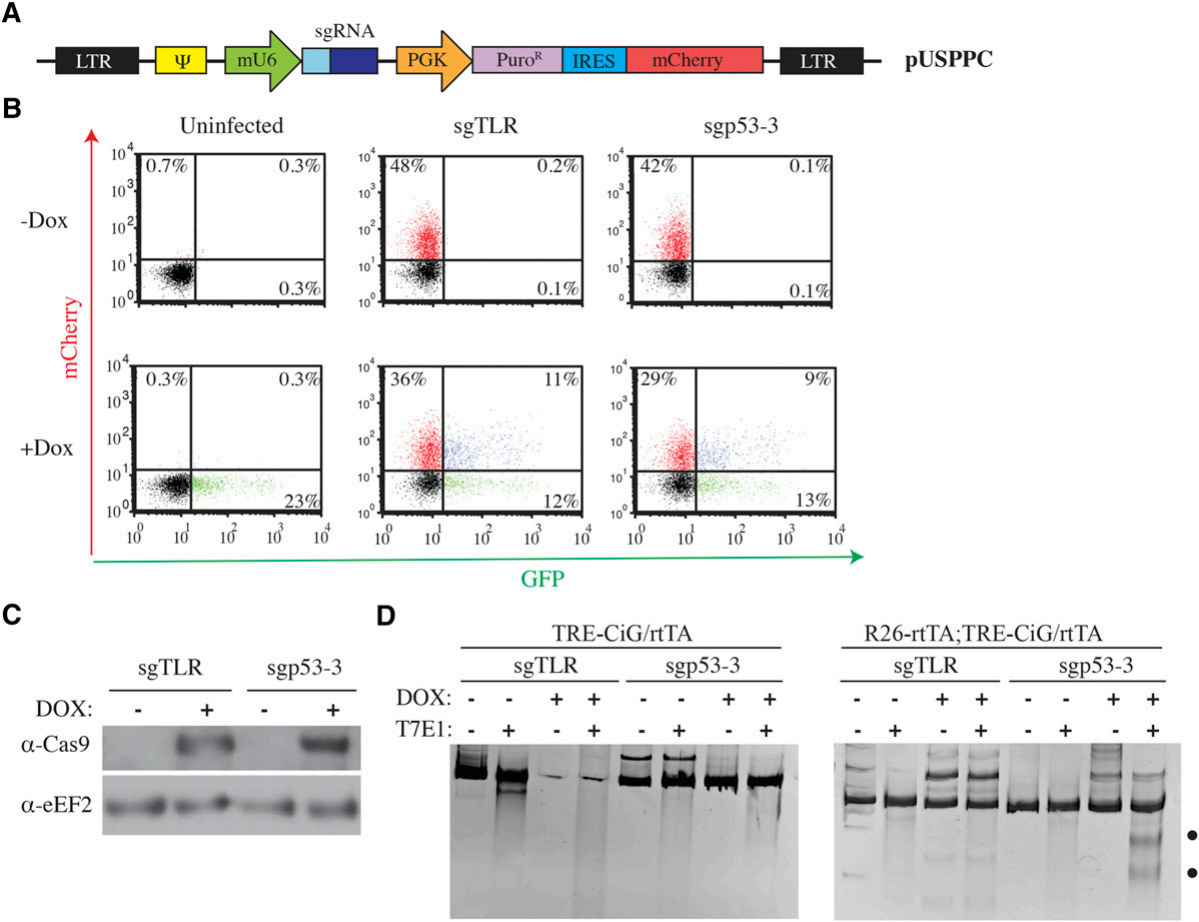
DOX-inducible genome editing *ex vivo* in HSPCs. A. Schematic representation of pUSPPC. LTR, long terminal repeat; Ψ, retroviral psi packaging element; mU6, murine U6 promoter; sgRNA, single guide RNA; PGK, phosphoglycerate kinase promoter; PuroR, puromycin resistance protein; IRES, internal ribosome entry site. B. Flow cytometry analysis of R26-rtTA;TRE-CiG/rtTA HSPCs infected with pUSPPC expressing a neutral sgRNA (sgTLR: ^5′^AGCAGCGTCTTCGAGAGTG^3′^) or one targeting p53 (sgp53-3: ^5′^AAGUCACAGCACAUGACGG^3′^) (Malina *et al.* 2013). Following infection, cells were exposed to vehicle or DOX (1 μg/ml) for 3 days. Infected cells are mCherry^+^ and those responsive to DOX are mCherry^+^/GFP^+^. C. Western blot showing induction of Cas9 expression in R26-rtTA;TRE-CiG/rtTA HSPCs exposed to DOX for 3 days. D. T7E1 assay from DNA isolated from the indicated HSPCs. Black dots denote the position of migration of cleaved products observed with DNA from pUSPPC-sgp53-3 infected R26-rtTA;TRE-CiG/rtTA HSPCs exposed to DOX for 2 days.

As a prelude to future experiments in which sgRNA libraries could be used for *in vivo* screens in the Eμ-Myc model (Wendel *et al.* 2007), we sought to assess if we could obtain Cas9-mediated editing in HSPCs that had been isolated from R26-rtTA;TRE-CiG/rtTA mice. For this, we tailored a retroviral delivery vector, pUSPPC, to constitutively express sgRNAs as well as puromycin and mCherry selectable markers (Figure 4A). Transduction of HSPCs derived from R26-rtTA;TRE-CiG/rtTA mice with pUSPPC lead to infection rates ranging from 38–48% (mCherry^+^ cells) (Figure 4B). Cas9 expression was induced upon exposure of HSPCs to DOX *ex vivo* (Figure 4C). Infection of these HSPCs with pUSPPC-sgp53-3 resulted in editing at the p53 locus, as assessed by the T7E1 cleavage assay (Figure 4D). This was not observed with HSPCs infected with pUSPPC-sgpTLR, a retrovirus expressing a neutral control sgRNA. Consistent with our finding that higher levels of Cas9 are induced in the presence of two rtTA alleles (Figure 2), higher levels of modification were observed in HSPCs derived from R26-rtTA;TRE-CiG/rtTA mice compared to HSPCS from TRE-CiG/rtTA mice (Figure 4D). These results demonstrate that Cas9 is inducible and functional in HSPCs and that conditional editing can be achieved *ex vivo* in HSPCs.

### Ex vivo manipulation of HSPCs and adoptive transfer experiment in the Eµ-Myc GEMM

**Figure 5:**
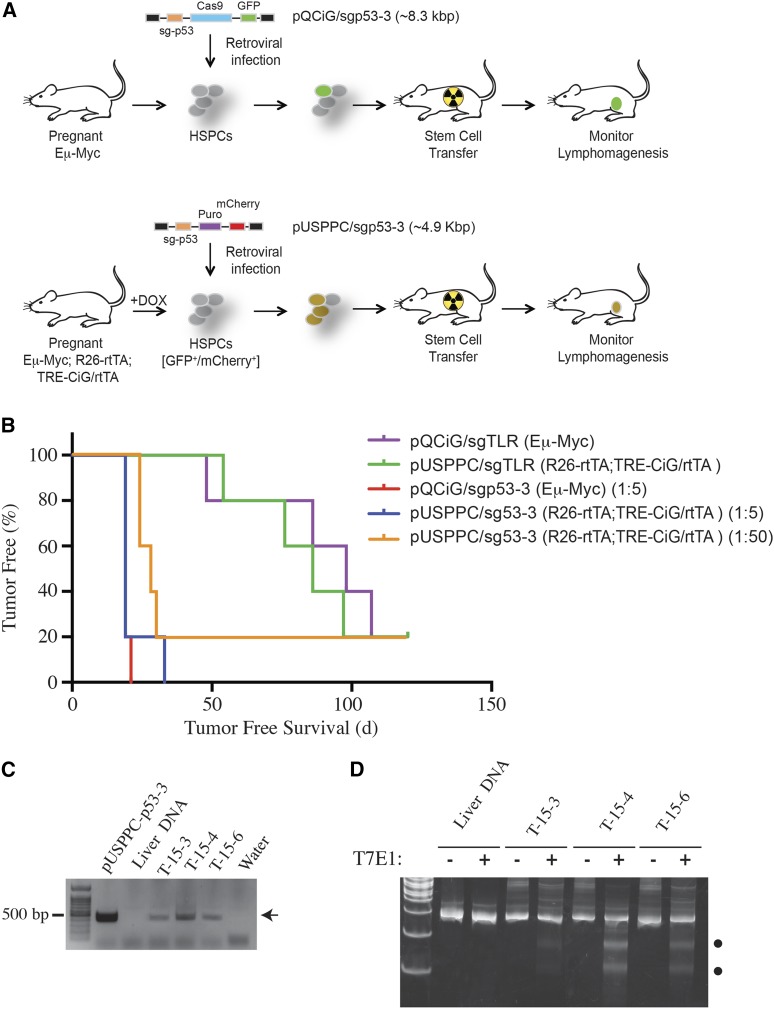
Assessing sgp53-3 oncogenic activity *in vivo* of different sgRNA pools. A. Schematic representation of stem cell transplant experiments involving Eμ-Myc HSPCs infected with pQCiG/sgp53-3 or Eμ-Myc; R26-rtTA; TRE-CiG/rtTA HSPCs infected with pUSPPC/sgp53-3. B. Kaplan-Meier curve illustrating tumor-free survival of C57Bl/6 mice transplanted with Cas9-modified HSPCs. Eµ-Myc HSPCs were transduced with pQCiG/sgTLR or a 1:5 dilution of pQCiG/sgp53-3 with pQCiG/sgTLR. Eµ-Myc;R26-rtTA;TRE-CiG/rtTA HSPCs were transduced with pUSPPC/sgTLR or 1:5 and 1:50 dilutions of pUSPPC/sgp53-3 with pUSPPC/sgTLR. N = 5 mice for each cohort. C. Recovery of sg53-3 sequences from tumors (T-15-3, T-15-4, T-15-6) arising from pUSPPC/sgp53-3 infected Eµ-Myc;R26-rtTA;TRE-CiG/rtTA HSCs. pUSPPC-p53-3 plasmid served as positive control and liver genomic DNA and water input served as negative controls. PCR products were analyzed on a 0.8% agarose gel. Arrowhead indicates expected location of migration of PCR products. D. T7E1 assay from DNA isolated from the indicated samples. Black dots denote the position of migration of cleaved products. The presence of undigested product is likely the consequence of contaminating normal cells during tissue extraction.

We have previously demonstrate that co-delivery of Cas9 with an sgRNA targeting p53 (pQCiG/sgp53-3) in Eµ-Myc HSPCs *ex vivo* accelerated tumor onset rates in transplanted normal, syngeneic recipients (Malina *et al.* 2013). However, transduction efficiencies of HSPCs with the All-In-One pQCiG/sgp53-3vector was low – likely a consequence of the large vector size required to deliver both Cas9 and the sgRNA, a feature known to negatively influence packaging and retroviral titers (Gelinas and Temin 1986). This limitation precludes using high complexity pools during genetic screens (Katigbak *et al.* 2016). In fact, in a previously reported screen involving HSPCs and pQCiG to identify oncogenic drivers in Burkitt’s lymphoma, we were restricted to using pools of only 5 sgRNAs since pools of 20 sgRNAs failed to identify p53 as an oncogenic driver (Katigbak *et al.* 2016). We reasoned that the smaller size of pUSPPC would allow for higher pool complexities in such *in vivo* screens. Infection of R26-rtTA;TRE-CiG/rtTA;Eµ-Myc with pUSPPC driving an sgp53-3, following by stem cell transplant and monitoring for lymphomagenesis revealed that dilutions of pUSPPC/sgp53-3 up to 1:50 were still capable of yielding tumors (Figure 5A, B). Analysis of three tumors arising from pUSPPC/sg53-3 infected R26-rtTA;TRE-CiG/rtTA;Eµ-Myc HSCs revealed that we could recover the sgp53-3 sequences by PCR from the tumor sample (Figure 5C) and that mutations at the targeted locus had arisen (Figure 5D). These results demonstrate that engineered Cas9 expression in target cells of interest will facilitate *in vivo* genetic screens by enabling the use of higher complexity pools.

## Discussion

Here, we use RCME to generate a mouse model that expresses Cas9 in an inducible manner across a wide range of tissues. RCME is a powerful approach by which to generate Cas9 transgenic mice as it enables the insertion of Cas9 into a well-characterized locus, thus providing predictable and reproducible expression. The development of orthogonal Cas9 editing tools, as well as the extension of RNA-guided DNA targeting of catalytically inactive Cas9-fusions to study transcriptional activation, transcriptional repression, epigenetic modification, base editing, and locus imaging have greatly increased the range of applications of the CRISPR-Cas9 system (Sternberg and Doudna 2015). We envisage that RCME will be a powerful way to make additional transgenic models that allow expansion of the CRISPR-Cas9 tool set beyond genome engineering.

Although Cas9-expressing mice exist, our iteration distinguishes itself in several ways. Platt *et al.* (Platt *et al.* 2014) developed a Cre-dependent CRISPR-Cas9 mouse which constitutively expresses Cas9 in tissues expressing Cre-recombinase. Although in their model (as in ours) long-term Cas9 expression was not deleterious at an organismal level, Cas9 is a foreign antigen and one concern is the generation of an immune response that could result in elimination of cells from the edited pool. Indeed, experiments with adenovirus-mediated delivery of Cas9 and sgRNA to the liver uncovered a Cas9-specific immune response (Wang *et al.* 2015). Similar findings were also reported following delivery of split-Cas9 moieties via adeno-associated virus (AAV) vectors (Chew *et al.* 2016). As well, given that off-target effects are an ever-present concern when it comes to genome editing, constitutive Cas9 expression is less desirable since longer-term Cas9 expression is associated with more off-target damage than transient Cas9 expression (Do *et al.* 2014; Zuris *et al.* 2015). Our system allows for controlled, short-term induction of Cas9 expression, which should decrease off-target mutagenesis and mitigate host immune responses against Cas9. There are several features of our system that could improve editing efficiency which would need to be systematically assessed. For example, editing in adoptive transplant experiments may benefit from extending the DOX treatment to recipient mice to sustain prolonged Cas9 expression. Dow *et al.* (Dow *et al.* 2015) have successfully generated transgenic mice that demonstrate inducible CRISPR/Cas9 editing upon DOX administration. This inducible system is quite powerful but limited in that the sgRNA expression cassette is co-integrated with Cas9, therefore necessitating generation of a new strain for every target. The TRE-CiG/rtTA mice we describe here allows for greater flexibility to study genotype-phenotype relationships *in vivo*.

## Supplementary Material

Supplemental material is available online at www.g3journal.org/lookup/suppl/doi:10.1534/g3.117.300327/-/DC1.
