# Supplementary material for: Inducible Genome Editing with Conditional CRISPR/Cas9 Mice
Source: G3 (Bethesda). 2018 Mar 8;8(5):1627–35. doi: 10.1534/g3.117.300327 (PMC5940154; doi:10.1534/g3.117.300327)
Supplement: Supplementary file 1 [file 1627FigureS1.docx]

**Supplemental Figure 1.**

**Nucleotide sequence of pCol-TCiG-rtTA3 targeting fragment**

PGK promoter

ATG FRT

SAdpA

TetO Promoter

Flag-NLS-Cas9 – UPPERCASE

EMCV IRES

GFP

RBGpA

CAGGs Promoter

rtTA3

SV40pA

ctctagaactagtggatcccccgggctgcaggaattaattcgtcgacctcgaaattctaccgggtaggggaggcgcttttcccaaggcagtctggagcatgcgctttagcagccccgctgggcacttggcgctacacaagtggcctctggcctcgcacacattccacatccaccggtaggcgccaaccggctccgttctttggtggccccttcgcgccaccttctactcctcccctagtcaggaagttcccccccgccccgcagctcgcgtcgtgcaggacgtgacaaatggaagtagcacgtctcactagtctcgtgcagatggacagcaccgctgagcaatggaagcgggtaggcctttggggcagcggccaatagcagctttgctccttcgctttctgggctcagaggctgggaaggggtgggtccgggggcgggctcaggggcgggctcaggggcggggcgggcgcccgaaggtcctccggaggcccggcattctgcacgcttcaaaagcgcacgtctgccgcgctgttctcctcttcctcatctccgggcctttcgacctgcatccatctag**atctcgatcgagcagctgaag**cttatggagaagttactattccgaagttcctattctctagaaagtataggaacttcgatccccctcgatcccccgggctgcagatctgtagggcgcagtagtccagggtttccttgatgatgtcatacttatcctgtcccttttttttccacagctcgcggttgaggacaaactcttcgcggtctttccagtggggatcgacggtatcgataagcttgcagatctgcgactctagaggatctgcgactctagaggatcataatcagccataccacatttgtagaggttttacttgctttaaaaaacctcccacacctccccctgaacctgaaacataaaatgaatgcaattgttgttgttaacttgtttattgcagcttataatggttacaaataaagcaatagcatcacaaatttcacaaataaagcatttttttcactgcattctagttgtggtttgtccaaactcatcaatgtatcttatcatgtctggatctgcgactctagaggatcataatcagccataccacatttgtagaggttttacttgctttaaaaaacctcccacacctccccctgaacctgaaacataaaatgaatgcaattgttgttgttaacttgtttattgcagcttataatggttacaaataaagcaatagcatcacaaatttcacaaataaagcatttttttcactgcattctagttgtggtttgtccaaactcatcaatgtatcttatcatgtctggatcgagtttaccactccctatcagtgatagagaaaagtgaaagtcgagtttaccactccctatcagtgatagagaaaagtgaaagtcgagtttaccactccctatcagtgatagagaaaagtgaaagtcgagtttaccactccctatcagtgatagagaaaagtgaaagtcgagtttaccactccctatcagtgatagagaaaagtgaaagtcgagtttaccactccctatcagtgatagagaaaagtgaaagtcgagtttaccactccctatcagtgatagagaaaagtgaaagtcgagctcggtacccgggtcgaggtaggcgtgtacggtgggaggcctatataagcagagctcgtttagtgaaccgtcagatcgcctggagacgccatccacgctgttttgacctccatagaagacaccgggaccgatccagcctccgcggccccgaattgaccaccATGGTGATGCATATGGCCGTGAAGAGACCCGCCGCCACCAAGAAGGCCGGCCAGGCCAAGAAGAAGAAGCTGGATTATAAAGATCATGATGGCGATTATAAAGATCATGATATTGATTATAAAGATGATGATGATAAAATGGCGCCGAAAAAAAAACGCAAAGTGAACATGGACAAGAAGTACAGCATCGGCCTGGACATCGGTACCAACAGCGTGGGCTGGGCCGTGATCACC**GACGAGTACAAGGTGCCTAG**CAAGAAGTTCAAGGTGCTGGGCAACACCGACAGGCACAGCATCAAGAAGAACCTGATCGGCGCCCTGCTGTTCGACAGCGGCGAGACCGCCGAGGCCACCAGGCTGAAGAGGACCGCCAGGAGGAGGTATACCAGGAGGAAGAACAGGATCTGCTACCTGCAGGAGATTTTCAGCAACGAGATGGCCAAGGTGGACGACAGCTTCTTCCACCGGCTGGAGGAGAGCTTCCTGGTGGAGGAGGACAAGAAGCACGAGAGGCACCCTATCTTCGGCAACATCGTGGACGAGGTGGCCTACCACGAGAAGTACCCTACCATCTACCACCTGAGGAAGAAGCTGGTGGACAGCACCGACAAGGCCGACCTGAGGCTGATCTACCTGGCCCTGGCCCACATGATCAAGTTCAGGGGCCACTTCCTGATCGAGGGCGACCTGAACCCTGACAACAGCGACGTGGACAAGCTGTTCATCCAGCTGGTGCAGACCTACAACCAGCTGTTCGAGGAGAACCCTATCAACGCCAGCGGCGTGGACGCCAAGGCCATCCTGAGCGCCAGGCTGAGCAAGAGCAGGAGGCTGGAGAACCTGATCGCCCAGCTGCCTGGCGAGAAGAAGAACGGCCTGTTCGGCAACCTGATCGCCCTGAGCCTGGGCCTGACCCCTAACTTCAAGAGCAACTTCGACCTGGCCGAGGACGCCAAGCTGCAGCTGAGCAAGGACACCTACGACGACGACCTGGACAACCTGCTGGCCCAGATCGGCGACCAGTACGCCGACCTGTTCCTGGCCGCCAAGAACCTGAGCGACGCCATCCTGCTGAGCGACATCCTGAGGGTGAACACCGAGATCACCAAGGCCCCTCTGAGCGCCAGCATGATCAAGAGATACGACGAGCACCACCAGGACCTGACCCTGCTGAAGGCCCTGGTCAGGCAGCAGCTGCCTGAGAAGTACAAGGAGATTTTCTTCGACCAGAGCAAGAACGGCTACGCCGGCTACATCGACGGCGGCGCCAGCCAGGAGGAGTTCTACAAGTTCATCAAGCCTATCCTGGAGAAGATGGACGGCACCGAGGAGCTGCTGGTGAAGCTGAACAGGGAGGACCTGCTGAGGAAGCAGAGGACCTTCGACAACGGCAGCATCCCTCACCAGATCCACCTGGGCGAGCTGCACGCCATCCTGAGGAGGCAGGAGGACTTCTACCCTTTCCTGAAAGACAACAGGGAGAAGATCGAGAAGATCCTGACCTTCAGGATTCCTTACTACGTGGGCCCTCTGGCCCGGGGCAACAGCAGATTCGCCTGGATGACCAGGAAGAGCGAGGAGACCATCACCCCTTGGAACTTCGAGGAGGTGGTGGACAAGGGCGCCAGCGCCCAGAGCTTCATCGAGAGGATGACCAACTTCGACAAGAACCTGCCTAACGAGAAGGTGCTGCCTAAGCACAGCCTGCTGTACGAGTACTTCACCGTGTACAACGAGCTGACCAAGGTGAAATACGTGACCGAGGGCATGAGGAAGCCTGCCTTCCTGAGCGGCGAGCAGAAGAAGGCCATCGTGGACCTGCTGTTCAAGACCAACAGGAAGGTGACCGTGAAGCAGCTGAAGGAGGACTACTTCAAGAAGATCGAGTGCTTCGACAGCGTGGAGATCAGCGGCGTGGAGGACAGGTTCAACGCCAGCCTGGGCACCTACCACGACCTGCTGAAGATCATCAAGGACAAGGACTTCCTGGACAACGAGGAGAACGAGGACATCCTGGAGGACATCGTGCTGACCCTGACCCTGTTCGAGGACAGGGAGATGATCGAGGAGAGGCTGAAGACCTACGCCCACCTGTTCGACGACAAGGTGATGAAGCAGCTGAAGAGGAGGAGATACACCGGCTGGGGCAGGCTGAGCAGGAAGCTGATCAACGGCATCAGGGACAAGCAGAGCGGCAAGACCATCCTGGACTTCCTGAAGAGCGACGGCTTCGCCAACAGGAACTTCATGCAGCTGATCCACGACGACAGCCTGACCTTCAAGGAGGACATCCAGAAGGCCCAGGTGAGCGGCCAGGGCGACAGCCTGCACGAGCACATCGCCAACCTGGCCGGCAGCCCTGCCATCAAGAAGGGCATCCTGCAGACCGTGAAGGTGGTGGACGAGCTGGTGAAGGTGATGGGCAGGCACAAGCCTGAGAACATCGTGATCGAGATGGCCAGGGAGAACCAGACCACCCAGAAGGGCCAGAAGAACAGCAGGGAGAGGATGAAGAGGATCGAGGAGGGCATCAAGGAGCTGGGCAGCCAGATCTTGAAGGAGCACCCTGTGGAGAACACCCAGCTGCAGAACGAGAAGCTGTACCTGTACTACCTGCAAAACGGCAGGGACATGTACGTGGACCAGGAGCTGGACATCAACAGGCTGAGCGACTACGACGTGGACCACATCGTGCCTCAGAGCTTCCTGAAGGACGACAGCATCGACAACAAGGTGCTGACCAGAAGCGACAAGAACAGGGGCAAGAGCGACAACGTGCCTAGCGAGGAGGTGGTGAAGAAGATGAAGAACTACTGGAGGCAGCTGCTGAACGCCAAGCTTATCACCCAGAGGAAGTTCGACAACCTGACCAAGGCCGAGAGGGGCGGCCTGAGCGAGCTGGACAAGGCCGGCTTCATCAAGAGGCAGCTGGTGGAGACCAGGCAGATCACCAAGCACGTGGCCCAGATCCTGGACAGCAGGATGAACACCAAATACGACGAGAACGACAAGCTGATCAGGGAGGTGAAGGTGATCACCCTGAAGAGCAAGCTGGTGAGCGACTTCAGGAAGGACTTCCAGTTCTACAAGGTGAGGGAGATCAACAACTACCACCACGCCCACGACGCCTACCTGAACGCCGTGGTGGGCACCGCCCTGATCAAGAAGTACCCTAAGCTGGAGAGCGAGTTCGTGTACGGCGACTACAAGGTGTACGACGTGAGGAAGATGATCGCCAAGAGCGAGCAGGAGATCGGCAAGGCCACCGCCAAGTACTTCTTCTACTCCAACATCATGAACTTCTTCAAGACCGAGATCACCCTGGCCAACGGCGAGATCAGGAAGAGACCTCTGATCGAGACCAACGGCGAGACCGGCGAGATCGTGTGGGACAAGGGCAGGGACTTCGCCACCGTGAGGAAGGTGCTGTCAATGCCTCAGGTGAACATCGTGAAGAAGACCGAGGTGCAGACCGGCGGCTTCAGCAAGGAGAGCATCCTGCCTAAGAGGAACAGCGACAAGCTGATCGCCAGGAAGAAGGACTGGGACCCTAAGAAGTACGGCGGCTTCGACAGCCCTACCGTGGCCTACAGCGTGCTGGTGGTGGCCAAGGTGGAGAAGGGCAAGAGCAAGAAGCTGAAGAGCGTGAAGGAGCTGCTGGGCATCACCATCATGGAGAGGAGCAGCTTCGAGAAGAACCCTATCGACTTCCTGGAGGCCAAGGGCTACAAGGAGGTGAAGAAGGACCTGATCATCAAGCTGCCTAAGTACAGCCTGTTCGAGCTGGAGAACGGCAGGAAGAGGATGCTGGCCAGCGCCGGCGAGCTGCAGAAGGGCAACGAGCTGGCCCTGCCTAGCAAATACGTGAACTTCCTGTACCTGGCCAGCCACTACGAGAAGCTGAAGGGCAGCCCTGAGGACAACGAGCAGAAGCAGCTGTTCGTGGAGCAGCACAAGCACTACCTGGACGAGATCATCGAGCAGATCAGCGAGTTCAGCAAGAGGGTGATCCTGGCCGACGCCAACCTGGACAAGGTGCTGAGCGCCTACAACAAGCACAGGGACAAGCCTATCAGGGAGCAGGCCGAGAACATCATCCACCTGTTCACCCTGACCAACCTGGGCGCCCCTGCCGCCTTCAAGTACTTCGACACCACCATCGA**CAGGAAGAGATACACCAGCA**CCAAGGAGGTGCTGGACGCCACCCTGATCCACCAGAGCATCACCGGCCTGTACGAGACCAGGATCGACCTGAGCCAGCTGGGCGGCGACTGAgatatcgcctgctaagtaaacgtcatttctaaggccaaaggctcttttcagagccaccccattaatcgatttgaattccgccccccccccctaacgttactggccgaagccgcttggaataaggccggtgtgcgtttgtctatatgttattttccaccatattgccgtcttttggcaatgtgagggcccggaaacctggccctgtcttcttgacgagcattcctaggggtctttcccctctcgccaaaggaatgcaaggtctgttgaatgtcgtgaaggaagcagttcctctggaagcttcttgaagacaaacaacgtctgtagcgaccctttgcaggcagcggaaccccccacctggcgacaggtgcctctgcggccaaaagccacgtgtataagatacacctgcaaaggcggcacaaccccagtgccacgttgtgagttggatagttgtggaaagagtcaaatggctctcctcaagcgtattcaacaaggggctgaaggatgcccagaaggtaccccattgtatgggatctgatctggggcctcggtgcacatgctttacatgtgtttagtcgaggttaaaaaacgtctaggccccccgaaccacggggacgtggttttcctttgaaaaacacgatgataatatggccacaaccatggtgagcaagggcgaggagctgttcaccggggtggtgcccatcctggtcgagctggacggcgacgtaaacggccacaagttcagcgtgtccggcgagggcgagggcgatgccacctacggcaagctgaccctgaagttcatctgcaccaccggcaagctgcccgtgccctggcccaccctcgtgaccaccctgacctacggcgtgcagtgcttcagccgctaccccgaccacatgaagcagcacgacttcttcaagtccgccatgcccgaaggctacgtccaggagcgcaccatcttcttcaaggacgacggcaactacaagacccgcgccgaggtgaagttcgagggcgacaccctggtgaaccgcatcgagctgaagggcatcgacttcaaggaggacggcaacatcctggggcacaagctggagtacaactacaacagccacaacgtctatatcatggccgacaagcagaagaacggcatcaaggtgaacttcaagatccgccacaacatcgaggacggcagcgtgcagctcgcc**gaccactaccagcagaacacc**cccatcggcgacggccccgtgctgctgcccgacaaccactacctgagcacccagtccgccctgagcaaagaccccaacgagaagcgcgatcacatggtcctgctggagttcgtgaccgccgccgggatcactctcggcatggacgagctgtacaagtaatgaattaatTAAACGCGTTGAGAACTTCAGGGTGAGTTTGGGGACCCTTGATTGTTCTTTCTTTTTCGCTATTGTAAAATTCATGTTATATGGAGGGGGCAAAGTTTTCAGGGTGTTGTTTAGAATGGGAAGATGTCCCTTGTATCACCATGGaccctcatgataattttgtttctttcactttctactctgttgacaaccattgtctcctcttattttcttttcattttctgtaactttttcgttaaactttagcttgcatttgtaacgaatttttaaattcacttttgtttatttgtcagattgtaagtactttctctaatcacttttttttcaaggcaatcagggtatattatattgtacttcagcacagttttagagaacaattgttataattaaatgataaggtagaatatttctgcatataaattctggctggcgtggaaatattcttattggtagaaacaactacaccctggtcatcatcctgcctttctctttatggttacaatgatatacactgtttgagatgaggataaaatactctgagtccaaaccgggcccctctgctaaccatgttcatgccttcttctctttcctacagctcctgggcaacgtgctggttgttgtgctgtctcatcattttggcaaaggattcactcctcaggtgcaggctgcctatcagaaggtggtggctggtgtggccaatgccctggctcacaaataccactgagatcgttttccctctgccaaaaattatggggacatcatgaagccccttgagcatctgacttctggctaataaaggaaatttattttcattgcaatagtgtgttggaatttctcgatcgctagcatcctaggcgcgccctattaatagtaatcaattacggggtcattagttcatagcccatatatggagttccgcgttacataacttacggtaaatggcccgcctggctgaccgcccaacgacccccgcccattgacgtcaataatgacgtatgttcccatagtaacgccaatagggactttccattgacgtcaatgggtggactatttacggtaaactgcccacttggcagtacatcaagtgtatcatatgccaagtacgccccctattgacgtcaatgacggtaaatggcccgcctggcattatgcccagtacatgaccttatgggactttcctacttggcagtacatctacgtattagtcatcgctattaccatgcgtcgaggtgagccccacgttctgcttcactctccccatctcccccccctccccacccccaattttgtatttatttattttttaattattttgtgcagcgatgggggcggggggggggggggcgcgcgccaggcggggcggggcggggcgaggggcggggcggggcgaggcggagaggtgcggcggcagccaatcagagcggcgcgctccgaaagtttccttttatggcgaggcggcggcggcggcggccctataaaaagcgaagcgcgcggcgggcgggagtcgctgcgttgccttcgccccgtgccccgctccgcgccgcctcgcgccgcccgccccggctctgactgaccgcgttactcccacaggtgagcgggcgggacggcccttctcctccgggctgtaattagcgcttggtttaatgacggctcgtttcttttctgtggctgcgtgaaagccttaaagggctccgggagggccctttgtgcgggggggagcggctcggggggtgcgtgcgtgtgtgtgtgcgtggggagcgccgcgtgcggcccgcgctgcccggcggctgtgagcgctgcgggcgcggcgcggggctttgtgcgctccgcgtgtgcgcgaggggagcgcggccgggggcggtgccccgcggtgcgggggggctgcgaggggaacaaaggctgcgtgcggggtgtgtgcgtgggggggtgagcagggggtgtgggcgcggcggtcgggctgtaacccccccctgcacccccctccccgagttgctgagcacggcccggcttcgggtgcggggctccgtgcggggcgtggcgcggggctcgccgtgccgggcggggggtggcggcaggtgggggtgccgggcggggcggggccgcctcgggccggggagggctcgggggaggggcgcggcggccccggagcgccggcggctgtcgaggcgcggcgagccgcagccattgccttttatggtaatcgtgcgagagggcgcagggacttcctttgtcccaaatctggcggagccgaaatctgggaggcgccgccgcaccccctctagcgggcgcgggcgaagcggtgcggcgccggcaggaaggaaatgggcggggagggccttcgtgcgtcgccgcgccgccgtccccttctccatctccagcctcggggctgccgcagggggacggctgccttcgggggggacggggcagggcggggttcggcttctggcgtgtgaccggcggctctagagcctctgctaaccatgttcatgccttcttctttttcctacagctcctgggcaacgtgctggttgttgtgctgtctcatcattttggcaaagagagctcactagtggtaccatgagtagactggacaagagcaaagtcataaacggagctctggaattactcaatggtgtcggtatcgaaggcctgacgacaaggaaactcgctcaaaagctgggagttgagcagcctaccctgta**ctggcacgtgaagaacaagcgg**gccctgctcgatgccctgccaatcgagatgctggacaggcatcatacccacttctgccccctggaaggcgagtcatggcaagactttctgcggaacaacgccaagtcataccgctgtgctctcctctcacatcgcgacggggctaaagtgcatctcggcacccgcccaacagagaaacagtacgaaaccctggaaaatcagctcgcgttcctgtgtcagcaaggcttctccctggagaacgcactgtacgctctgtccgccgtgggccactttacactgggctgcgtattggaggaacaggagcatcaagtagcaaaagaggaaagagagacacctaccaccgattctatgcccccacttctgagacaagcaattgagctgttcgaccggcagggagccgaacctgccttccttttcggcctggaactaatcatatgtggcctggagaaacagctaaagtgcgaaagcggcgggccgaccgacgcccttgacgattttgacttagacatgctcccagccgatgcccttgac**gactttgaccttgatatgctg**cctgctgacgctcttgacgattttgaccttgacatgctccccgggtaatcgcgaatgcatctagatatcggatccataactgatcataatcagccataccacatttgtagaggttttacttgctttaaaaaacctcccacacctccccctgaacctgaaacataaaatgaatgcaattgttgttgttaacttgtttattgcagcttataatggttacaaataaagcaatagcatcacaaatttcacaaataaagcatttttttcactgcattctagttgtggtttgtccaaactcatcaatgtatcttaggcgcgcccgcaagcttggcgtaatcatggtcatagctgtttcctgtgtgaaattgttatccgctcacaattccacacaacatacgagccggaagcataaagtgtaaagcctggggtgcc….
