# Supplementary material for: Inducible Genome Editing with Conditional CRISPR/Cas9 Mice
Source: G3 (Bethesda). 2018 Mar 8;8(5):1627–35. doi: 10.1534/g3.117.300327 (PMC5940154; doi:10.1534/g3.117.300327)
Supplement: Supplementary file 2 [file 1627FigureS2.docx]

**Supplemental Figure 2.**

**Nucleotide sequence of pUSPPC**

Psi+

mU6 Promoter

sgRNA Cassette

PGK Promoter

Puromycin Resistant Marker

EMCV IRES

mCherry

SIN-LTR

gccagcaacttatctgtgtctgtccgattgtctagtgtctatgtttgatgttatgcgcctgcgtctgtactagttagctaactagctctgtatctggcggacccgtggtggaactgacgagttctgaacacccggccgcaaccctgggagacgtcccagggactttgggggccgtttttgtggcccgacctgaggaagggagtcgatgtggaatccgaccccgtcaggatatgtggttctggtaggagacgagaacctaaaacagttcccgcctccgtctgaatttttgctttcggtttggaaccgaagccgcgcgtcttgtctgctgcagcgctgcagcatcgttctgtgttgtctctgtctgactgtgtttctgtatttgtctgaaaattagggccagactgttaccactcccttaagtttgaccttaggtcactggaaagatgtcgagcggatcgctcacaaccagtcggtagatgtcaagaagagacgttgggttaccttctgctctgcagaatggccaacctttaacgtcggatggccgcgagacggcacctttaaccgagacctcatcacccaggttaagatcaaggtcttttcacctggcccgcatggacacccagaccaggtcccctacatcgtgacctgggaagccttggcttttgacccccctccctgggtcaagccctttgtacaccctaagcctccgcctcctcttcctccatccgccccgtctctcccccttgaacctcctcgttcgaccccgcctcgatcctccctttatccagccctcactccttctctaggcgccggaattgaagatcttctagagatccgacgccgccatctctaggcccgcgccggccccctcgcacagacttgtgggagaagctcggctactcccctgccccggttaatttgcatataatatttcctagtaactatagaggcttaatgtgcgataaaagacagataatctgttctttttaatactagctacattttacatgataggcttggatttctataagagatacaaatactaaattattattttaaaaaacagcacaaaaggaaactcaccctaactgtaaagtaattgtgtgttttgagactataaatatcaattggagaaaagccttgtttgagcagcgtcttcgagagtggttttagagctaggatcctagcaagttaaaataaggctagtccgttatcaacttgaaaaagtggcaccgagtcggtgctttttttctcgagcaaccagaattcaaggggctactttaggagcaattatcttgtttactaaaactgaataccttgctatctctttgatacatttttacaaagctgaattaaaatggtataaattaaatcacttttttcaattctaccgggtaggggaggcgcttttcccaaggcagtctggagcatgcgctttagcagccccgctgggcacttggcgctacacaagtggcctctggcctcgcacacattccacatccaccggtaggcgccaaccggctccgttctttggtggccccttcgcgccaccttctactcctcccctagtcaggaagttcccccccgccccgcagctcgcgtcgtgcaggacgtgacaaatggaagtagcacgtctcactagtctcgtgcagatggacagcaccgctgagcaatggaagcgggtaggcctttggggcagcggccaatagcagctttgctccttcgctttctgggctcagaggctgggaaggggtgggtccgggggcgggctcaggggcgggctcaggggcggggcgggcgcccgaaggtcctccggaggcccggcattctgcacgcttcaaaagcgcacgtctgccgcgctgttctcctcttcctcatctccgggcctttcgacctgcagcccaagcttaccatgaccgagtacaagcccacggtgcgcctcgccacccgcgacgacgtccccagggccgtacgcaccctcgccgccgcgttcgccgactaccccgccacgcgccacaccgtcgatccggaccgccacatcgagcgggtcaccgagctgcaagaactcttcctcacgcgcgtcgggctcgacatcggcaaggtgtgggtcgcggacgacggcgccgcggtggcggtctggaccacgccggagagcgtcgaagcgggggcggtgttcgccgagatcggcccgcgcatggccgagttgagcggttcccggctggccgcgcagcaacagatggaaggcctcctggcgccgcaccggcccaaggagcccgcgtggttcctggccaccgtcggcgtctcgcccgaccaccagggcaagggtctgggcagcgccgtcgtgctccccggagtggaggcggccgagcgcgccggggtgcccgccttcctggagacctccgcgccccgcaacctccccttctacgagcggctcggcttcaccgtcaccgccgacgtcgaggtgcccgaaggaccgcgcacctggtgcatgacccgcaagcccggtgcctgacgcccgccccacgacccgcagcgcccgaccgaaaggagcgcacgaccccatcatccaattccgccccccccccctaacgttactggccgaagccgcttggaataaggccggtgtgcgtttttaatatccccattaatcgatttgaattccgccccccccccctaacgttactggccgaagccgcttggaataaggccggtgtgcgtttgtctatatgttattttccaccatattgccgtcttttggcaatgtgagggcccggaaacctggccctgtcttcttgacgagcattcctaggggtctttcccctctcgccaaaggaatgcaaggtctgttgaatgtcgtgaaggaagcagttcctctggaagcttcttgaagacaaacaacgtctgtagcgaccctttgcaggcagcggaaccccccacctggcgacaggtgcctctgcggccaaaagccacgtgtataagatacacctgcaaaggcggcacaaccccagtgccacgttgtgagttggatagttgtggaaagagtcaaatggctctcctcaagcgtattcaacaaggggctgaaggatgcccagaaggtaccccattgtatgggatctgatctggggcctcggtgcacatgctttacatgtgtttagtcgaggttaaaaaacgtctaggccccccgaaccacggggacgtggttttcctttgaaaaacacgatgataatatggccacactaggcttttgcaaaaagctcatggtgagcaagggcgaggaggataacatggccatcatcaaggagttcatgcgcttcaaggtgcacatggagggctccgtgaacggccacgagttcgagatcgagggcgagggcgagggccgcccctacgagggcacccagaccgccaagctgaaggtgaccaagggtggccccctgcccttcgcctgggacatcctgtcccctcagttcatgtacggctccaaggcctacgtgaagcaccccgccgacatccccgactacttgaagctgtccttccccgagggcttcaagtgggagcgcgtgatgaacttcgaggacggcggcgtggtgaccgtgacccaggactcctccctgcaggacggcgagttcatctacaaggtgaagctgcgcggcaccaacttcccctccgacggccccgtaatgcagaagaagaccatgggctgggaggcctcctccgagcggatgtaccccgaggacggcgccctgaagggcgagatcaagcagaggctgaagctgaaggacggcggccactacgacgctgaggtcaagaccacctacaaggccaagaagcccgtgcagctgcccggcgcctacaacgtcaacatcaagttggacatcacctcccacaacgaggactacaccatcgtggaacagtacgaacgcgccgagggccgccactccaccggcggcatggacgagctgtacaagtaacctagtatcgatttgaattccgtagcaaaagaactgtttaaaccagctgaagcctatagagtacgagccatagataaaataaaagattttatttagtctccagaaaaaggggggaatgaaagaccccacctgtaggtttggcaagctagcttaagtaacgccattttgcaaggcatggaaaaatacataactgagaatagagaagttcagatcaaggtcaggaacagatggaacagggtcgaccctagagaaccatcagatgtttccagggtgccccaaggacctgaaatgaccctgtgccttatttgaactaaccaatcagttcgcttctcgcttctgttcgcgcgcttctgctccccgagctcaataaaagagcccacaacccctcactcggggcgccagtcctccgattgactgagtcgcccgggtacccgtgtatccaataaaccctcttgcagttgcatccgacttgtggtctcgctgttccttgggagggtctcctctgagtgattgactacccgtcagcgggggtctttcatt
